# Students' motivation in choosing general practice for their career pathway: a middle-income country report, Indonesia

**DOI:** 10.5116/ijme.6209.1974

**Published:** 2022-02-28

**Authors:** Hikmawati Nurokhmanti Hikma, Mora Claramita Mora, Ova Emilia Ova

**Affiliations:** 1 Department of Medical, Health Professions Education, and Bioethics, Faculty of Medicine, Public Health and Nursing, Universitas Gadjah Mada, Indonesia

**Keywords:** General Practitioner, postgraduate medical training, student career choice, Indonesia

## Abstract

**Objectives:**

To understand the medical students’ perspectives and influential factors on their career pathway decision to be a General Practitioners (GP) in Indonesia.

**Methods:**

This research used sequentially mixed methods. The qualitative study was conducted using focus group discussions with 30 third-year students, followed by in-depth interviews with 15 students from one Indonesian institution with the highest level of accreditation. The qualitative data, together with the literature review, were used to construct an online questionnaire with three types of questions.

**Results:**

The survey response rate reached 81% from 2,240 students across 64 faculties of medicine in Indonesia. Responses indicated that GP is not preferred as the leading career choice, and 67% of students prefer to become hospital specialists. The qualitative data revealed several influencing factors in choosing GP as their ultimate career choice: more family time, being closer to the community, and interest in bio-psycho-social subjects. Meanwhile, the reasons not to choose GP as a career choice were: imbalance in work and life, less authority, being at the lowest level position in the health care system, high uncertainty, and low financial incentives.

**Conclusions:**

GP is not an interesting career option for most medical students in this study. Considering GP works strategically in primary care settings aiming at better health outcomes and optimizing the health care financial system with greater patient satisfaction, influential positive factors to be GP should be nurtured in the medical curriculum.

## Introduction

Starfield (1998) defined the term primary care as an integrated and accessible health care service with clinicians who are capable of dealing with community health care needs, building sustainable relationships with patients, and practicing under family and community circumstances.^1^ This discipline requires specific clinical skills to manage and identify undifferentiated symptoms and match patients’ needs with health care resources.^2^ Those specific characteristics in primary health care services are essential for a general practitioner (GP) or family physician.

Over time, primary care services have demonstrated their positive impact on low- and middle-income populations.^3^ Moreover, a well-developed primary healthcare system with quality-minded General Practitioner is correlated with low mortality rates in almost all countries in the world.^4^ As a consequence, primary care services are essential for all countries, including the Indonesian health care system, which must deal with the triple burden of diseases: non-communicable and communicable diseases along with low to non-existent resources.^5^

In Indonesia, primary care service is not established as an expert in clinical career pathways. Indonesia has a population of 250 million, the fourth largest in the world, with 20,000 hospital specialists and more than 100,000 registered General Practitioner, who are not specialists but are graduates from 6 years of basic medical education. Essentially, those General Practitioner who work in primary health care mostly serves as ‘gate keepers’ to make appropriate referrals within the universal coverage system that began in 2014. Since General Practitioner works without any supervision and have only limited consultations with hospital specialists, primary care services from General Practitioners lead to lower-than-expected quality of service.

Recently, trends in the career choice to become a primary care physician have drastically declined in many countries such as Germany, Japan, and the United Kingdom.^6^^,^^7^^,^^8^ Although there are many established institutions already supporting the present number of General Practitioner, there are challenges to maintain an adequate number of General Practitioner in the future since it depends on sustained work of General Practitioner within the community and in prospective health care systems.

Several studies have explored the reasons why undergraduate students choose their career pathways. A systematic review of GP career trends divided the reasons into internal and external influences, which encourage or demotivate undergraduate students to select a career as a General Practitioner.^9^ Common encouraging reasons to work in primary care settings are: exposure to a rural or remote area, good role models, and working conditions. Simultaneously, common demotivating reasons are low income, low prestige, and the medical school environment that has greater trends toward hospital specialist training. Moreover, stress among General Practitioners, lack of procedures, loneliness in small work settings, and negative comments from hospital educators and specialists cause students to have low motivation to choose General Practitioner as their career.

Reasons students choose General Practitioner as their career pathway may differ between high- and low-income countries. In developed countries, intellectual challenges requiring a broad range of knowledge is a common reason not to become a General Practitioner. On the contrary, understanding of rural needs, attitude towards social problems, volunteer work, family influence, and shorter length of residency are common reasons to choose General Practitioner as a career pathway. In Saudi Arabia, female students tend to choose their career based on the possibility to get a scholarship and because of closer and more human interactions with patients. Considerably different, male students tend to choose a specialty with technical challenges, higher salaries, and greater opportunities for promotion and prestige.^10^ Whereas in Germany, where General Practitioner does not work as gatekeepers, General Practitioner are mostly females, older, unambitious concerning career possibilities, and prefer to have close physician-doctor relationships. This is because income and prestige are not their priorities.^6^

Motivation is an essential driver for students’ career choices. According to the motivation theory of self-determination by Ryan and Deci (1985), life decisions are based on a personification process. The self-determination theory of motivation proposes that motivation is a fluid and dynamic process which could change over time.^11^^,^^12^ Several factors influence students during their educational process, in which internal and external factors will become intertwined and shape the students’ ultimate motivation.^12^ This shaping process implies the importance of the educational system within each institution.^13^

Indonesia is a lower-middle-income country with unique multi-cultural characteristics in a developed universal health care system. There is no data on the Indonesian students’ career options yet. Furthermore, there is not much understanding on how medical student in lower middle income country choosing and reasoning on their career yet.^14^ This study aimed to understand the students’ perspectives and reasoning in choosing their career option in a middle-income country context which will be basic data in developing a General Practitioner specialist program for a better and sustainable Indonesian health care system.

## Methods

This exploratory study used a mixed-methods approach.^15^ First, a qualitative study was conducted in order to explore students’ perspectives and to construct an online questionnaire. Then, a quantitative study was conducted to confirm the qualitative data. This study was approved by the appropriate institutional ethics board, with approval letter number KE/FK/07652/EC/2017 published July 14, 2017. Participants’ written consent was also obtained after explaining the aim and procedures of this study prior to data collection.

### Qualitative study

The setting for the qualitative study was at the top medical school in Indonesia. From a total of 36 tutorial groups in third-year students, one student from every tutorial group was invited to participate in the Focus Group Discussion (FGDs). The invited students were randomly assigned to join the focus group discussion in each focus group discussion participated by six to nine students. The focus group discussion and in-depth interviews were done by using a structured interview guide as follows: (1) their opinion on health care systems, (2) future career choice, (3) their opinion about General Practitioner and selecting General Practitioner as their career pathway, and (4) how education/media are shaping their opinions on their career options. Additionally, fifteen students who had already finished their exit exam agreed to join the qualitative study for an in-depth interview with the principal investigator.

Data saturation was reached after the fourth focus group discussion. All focus group discussions and in-depth interviews were recorded, transcribed, and descriptively analyzed. The analysis process was divided into two phases: (1) content analysis of focus group discussion and in-depth interview transcripts, and (2) content analysis of the online survey questionnaire. The first content analysis results gave frame to the second analysis.^16^

The qualitative study themes were constructed based on the Focus Group Discussion and in-depth interview data. Afterwards, these items were categorized into a list of several grouped themes by two coders. The coders (the first and second author) worked individually on the open coding and had six meetings (one meeting per week for six weeks) to agree on the final themes. Discussion of the two coders was then validated by the last coder (the previous author) based on the literature framework about student motivation and influencing factors for choosing General Practice’s as a career choice.

### Quantitative study

The quantitative study was a national survey using an online questionnaire that was distributed to final year undergraduate students who had already finished all the curriculum, including clinical rotation, and had done the national exit exam to gain the professional practice license. The questionnaire was constructed based on the results of the previous qualitative study, and on a research report from a Swedish telehealth study conducted in Indonesia in 2017. Some modifications were done in the re-validation process by adding several questions based on a current literature review and previous qualitative data.

The final constructs of the online survey questionnaire were: (1) students’ characteristics, (2) students’ perspectives on a professional career, (3) students’ career-choice, (4) determining factors for future career choice, (5) preference in working style for future career choice and (6) educational impact on the career choice. Each part consisted of two to fourteen sub-questions and two short answer items.

Before starting the next part of the study, an intra-reliability test was done with 30 students by assessing the Cronbach alpha of each question. There were several questions with α Chronbach <0.70, which were then either dropped or modified. The final version was sent to our Information Technology office to be set into electronic format for the online survey questionnaire. An informed consent section was added in the beginning of the questionnaire, involving a statement that explained to students when clicking open the questionnaire that they agreed to join and complete this study. To determine the reliability of the resulting data, two border levels of missing data were used (above 5% or 10%).^17^

The answers of short questions from 1,778 online survey responses were condensed into 300 data items considering institution representational. We selected the 300 data points based on the random number of students per institution out of 64 institutions (25-35 results, randomly selected).

## Results

The overall results from the qualitative and quantitative studies showed that most students still had positive views concerning General Practitioner, but there were several reasons which made them have low motivation to choose to become General Practitioners as their career pathway, as represented below.

### Qualitative study

Overall, students said that General Practitioner is not their next future career goal in medicine. Becoming General Practitioner  would  only  be  a  temporary option  for  them before they choose specialties in medicine:

“GP….it is something positive but how to explain….it is positive as a starting career….” (FGD)

“Yes, it is a career option, it will be a phase which should be passed through, all of us should be a GP first” (In-depth interview)

The following reasons were mentioned for not choosing General Practitioner as their career pathway, including the science, working tasks/responsibilities, rewards, social hierarchy, and social/community perspectives, as reported below. 

### The scientific dimension of primary care

General Practitioner must have certain competencies which prepare them to fit into the present-day health care system. Regarding their role, General Practitioner are ideally prepared to make the best clinical decisions for their patients regarding their condition and to assist them during both times of good and poor health, as part of a continuity of care during a person’s lifetime. However, patient-centred care was not fully understood by most medical students, and they tended to be more focused on the development of medical knowledge.

“First, the science, then secondly also because of the previous one because the science flourish rapidly, getting more specialized even become sub-sub specialty….so with a condition like that, becoming GP just really needs to be upgraded” (FGD)

Working tasks/responsibilities

We found several interesting responses expressing dissatisfaction toward the General Practitioner they had met. Most students said that health consultations with General Practitioners were often too short and only based on symptoms which make the tasks of primary care not satisfying. Another main reason for their low motivation to become General Practitioner was the large numbers of patients to be seen within the limited time available. Furthermore, one respondent also indicated that General Practitioner were demanded to do more preventive tasks and speak diplomatically to their community which is hard according to some students.

“when my experiences met with GP…with my all achieved science, I think I could do more as a GP…” (FGD)

“From my personal perspective, I do not want to be a GP because GP only give symptomatic treatment. Only based on criteria, I even went to GP services three or four times, and I received the same medication, that make me underestimate them. That was experienced not only by me, but I also heard my friends had the same experiences, just like me, they also got so many medications, that’s why I do not want to be a GP” (FGD)

“If we go to a specialist, they will be more exploring into details, so that’s our impression, they are better, more precise” (FGD)

“…..GP should maintain their community health, so they should diplomatically speak to their community, and do more preventive tasks…”  (FGD)

### Rewards

For decades, General Practitioner accepted rewards from their patients, which came from patients’ out of pocket payments. They could only accept a limited amount based on the number of patients each day. The development of the national insurance system has greatly impacted the previous payment system. The new system pays the General Practitioner by a per-capita approach which enables them to accept salary-based income, regardless of the number of patients each day.

 “if seen from the salary point of view, it seems so little, although now we have capitation. Yesterday, it was said that the earning from the capita will be cut for operational costs of the unit and also for the head of the unit, the nurse….so the doctor will receive only a little” (In depth interview)

“From the PHC capitation, per patients they had 11 thousand rupiahs, and each PHC got around 10 thousand people, but I saw the doctor only receive 3 million 3 hundred rupiahs for one month, and I was really surprised because it is not enough for this time to live with that amount, I feel that I was not prepared. Then we should be a GP first, so it took a long time, and we are just worth that much” (FGD)

### Social hierarchy within the health care system

Several negative comments reflected the influence of social hierarchy on students’ perspectives of General Practitioner.

“’become a specialist! Why would you become a GP? You will regret it, just like me’……heard from a primary care doctor who complained like that, and was disappointed why they were not becoming a specialist” (In-depth interview)

### Social/community perspectives

In the past, under the previous health care system, General Practitioner had a special position in the community’s heart. However, recently together with the current universal health care system has caused many sociocultural changes within the community. General Practitioner providing primary care services are now seen as “the means to an end”, or as the main source of referrals to specialists. Some students provided the following evidence.

“Patients in my area are actually not in the city but in the village. In my village, our people tend to think why should I go to the GP because it will just cost their money twice if they already know the problem they just decide to go to the internist or pediatrics” (In-depth interview)

“The community trusts the specialists more, and have greater preference to go to specialist if they get ill which might be not necessarily like that because it might be that they only need GP level competency….” (FGD)

“My parents’ opinion is different, they see GP life is so miserable, and they see specialist is much better” (FGD)

“If just me, I do not have national insurance coverage, so if I want to have health care service, I will just go to specialist, if I go to GP, they will refer me anyway. So many just like me, if they got ill, they just go to the specialist. Also, besides they should have a long queue, I think that is why they choose to directly go to specialist” (In-depth interview)

We identified the same themes from the online survey questionnaire as we did with the first content analysis, which described the responses from the Focus Group Discussion and personal interviews, as illustrated in Table 1.

In the online survey, we found that 70% of the students are not interested in becoming a General Practitioner. We captured this phenomenon by a simple quote shared by many students who had low motivation and were not willing to become a General Practitioner.

“it is just because of low rewards, unspecific science, and big responsibility.” (e-survey)

### Quantitative study

From 2,204 students who took part in the national exit exam, 81% completed the online survey questionnaire with only a small percentage of missing data. There were 1,674 fully completed online survey questionnaires, predominantly by female students (60%), with 75% from urban areas and 87% not married yet.

In this study, each part of the quantitative analysis had some missing data, involving from 0.1% - 11%, except the identity part. Interestingly, data on ‘determining factors for their career pathway’ had below 5% missing data. As shown from the data pattern, when the students were asked about their preference of work, they did not give firm answers, but when asked about factors that influence the decision of their career pathway, they gave a firm answer. These contradictory patterns showed that the students were not really sure about their own working expectations and future career decisions.

**Table 1:** Qualitative result from the online survey

Coding categories - determining factors in choosing GP as their career path		What makes you interested to be a GP		What makes you not interested to be a GP?	
		Number of responses	Quotes example	Number of responses	Quotes example
1	Reward/salary	3	“enough rewards”	30	“unfair rewards between work with knowledge and working responsibilities” “low salary with minimal rewards”
2	The science	36	“various cases” “more experiences”	48	“too much to be learned” “too broad science”
3	Responsibility and competence, including · National assurance coverage (BPJS) · Time · Family time · Facility	32	“flexible working time” “front liners, first liner” “caring holistically”	40	“restricted facilities with limited competence” “under-controlled by assurance system” “took too much time for working”
4	Social hierarchy · cooperation · career	3		27	“community stigma” “unclear career pathway” “there too big gap between specialist and GP” “lower income than specialist”
5	Social/community perspectives:	40	“I Want to have a close relationship with the community” “Want to give service to the community.” “I want to be the first point of contact of the community.”	13	“low community interest” “having low trust from the community”
6	Spiritual	5	“Make good deed, achieve rewards.”	-	
Interested		36	Not interested	70	

**Table 2:** Students’ perspective on medical career (N=1674)

No	Item	1 (Strongly disagree) N (%)	2 (Disagree) N (%)	3 (Neutral) N (%)	4 (Agree) N (%)	5 (Strongly agree) N (%)
1	The need of specialty in working place	16(0.9)	20(1.1)	252(14.1)	671(37.5)	703(39.3)
2	Career as GP	100(5.6)	166(9.3)	763(42.7)	455(25.4)	175(9.8)
3	Career as doctor in remote area	69(3.9)	186(10.4)	747(41.8)	489(27.3)	161(9)
4	Career as specialist	18(1)	29(1.6)	389(21.8)	639(35.7)	588(32.9)

Students’ perspectives on their career in medicine in a working place are shown in Table 2. Most of the students (76%) agreed about having a specialty position at the place of work. Students had more positive perspectives on hospital specialists than General Practitioners. However, they had even more positive views on General practitioners working in remote areas. Choosing a specific specialty depends on personal interests, flexible working time, salary/rewards, and family time. Interestingly, working status, independence/responsibility, research opportunity, networking, serving the community, and career development were not determinant factors that tended to influence their choice of specialty, as shown in Table 3.

Preference of working type is one of the influential factors for students to choose their career pathway. As a result, students’ working type preference is dominated mainly by expectations for a positive work-life balance, using more technology, more procedural protocols, teamwork, involving not too complicated cases, and having more patient interaction.

The most significant external influencing factor on students’ determination of career pathway is their educational phase, as shown in Table 4. More than half (59%) of the students agreed that they had changed their perspective on becoming a General Practitioner after their education. However, they were prepared to be a General Practitioner or work in rural areas, as shown by 72% of responses. Also, 56% of the students were positively influenced by good General Practitioner role models.

**Table 3:** Factors determining in career pathway (N=1674)

No.	Item	Yes N (%)	No N (%)
6	Change perspective on General Practitioner after school	1057(59.1)	536(30)
1	Personal Interest	1088(60.9)	700(39.1)
2	Salary-rewards	942(52.7)	846(47.3)
3	Time for family	942(52.7)	846(47.3)
4	Flexible working time	937(52.4)	849(47.5)
5	To serve society	878(49.1)	910(50.9)
6	Career development	831(46.3)	957(53.3)
7	Leisure time	667(37.2)	1120(62.7)
8	Independence-responsibility	658(36.8)	1130(63.2)
9	Family distance	629(35.2)	1159(64.8)
10	Networking	613(34.3)	1175(65.7)
11	Opportunity to teach	482(27)	1306(73)
12	Working status	436(24.4)	1351(75.6)
13	Research opportunity	281(15.7)	1507(84.3)
14	Family with specialist career	206(11.5)	1582(88.5)

**Table 4:** Educational impact on students’ career determination (N=1674)

No	Item	Yes N (%)	No N (%)
1	Previous learning experiences on primary and secondary care settings directing work as General Practitioner.	1293(72.3)	307(17.2)
2	Previous learning experiences in remote areas clinical settings.	1005(56.2)	596(33.3)
3	Previous learning experiences with GP as a good role model.	1370(76.6)	221(12.4)
4	Previous learning experiences with other teachers’ positive comments on General Practitioner as positive role model in health care services.	1002(56)	589(32.9)
5	Previous learning experiences with other teachers comments on General Practitioners lack of role model within health care services.	535(29.9)	1059(59.2)

## Discussion

Data from this study indicate that General Practitioner is not a preferred career pathway for most medical students who are recently graduated. As expressed by one participant, ‘General Practitioner (GP) is not a destination but a destiny’. The students still have mostly positive views of General Practitioners, but they prefer to choose another career pathway. In line with their medical career pathway’s perceptions and preferences, most students plan to work as a GP for only 2-3 years. If there is a chance, they prefer to pursue a position as a hospital specialist or complete a master’s degree rather than staying as a General Practitioner for the rest of their career. This particular point of the results will influence the number of General Practitioners in Indonesia which are necessary for maintaining the current community health care system, especially in a country that has no graduate Family Medicine or General Practitioner specialist training. General Practitioners are essential in developing an effective and efficient health care system since they deal directly with the community and are on the front line in deciding the most effective care management for the people in their community.

Although students’ motivation to choose GP as their career pathway is low, students’ perspectives of General Practitioners is still a respectable career. Moreover, most of them also hope for a positive work-life balance. This balance is one strong reason which encourages medical students to choose GP as their career pathway.^8^^,^^11^ As a General Practitioner, working in primary care has specific characteristics which are influenced by personal style, i.e. health care practitioners serving their patients as a whole person (personalized-holistic care), practicing patient-centered medicine by giving consultations, allowing patients to express their perspectives, respecting patients’ authority in decision-making, and safeguarding self-autonomy.^18^ Patient-centered care medicine will require much more communication with patients, family, and community. Unfortunately, the advantages of life balanced with working as a General Practitioner is not recognized by many of the students as a significant benefit in working as health workers. Still, there is a strong chance for students to change their minds throughout their professional life.

In contrast, one strong reason for students not to choose General Practitioner as their career pathway is the low scientific character of the discipline as was also mentioned by Lambert (2012).^8^ Thus emphasis that students' expectation in working as health workers is dealing with only a specific science of medicine, while the roles of General Practitioners include taking care of all types of patients with different life phases which emphasises the need for broad medical knowledge.^18^ Furthermore, the reason for preferring a specific specialty by students could be caused by their lack of understanding about the actual nature of the General Practitioners, i.e. dealing with a complex patients’ life background and socio-determinants of health. Other reasons for their low motivation to become General Practitioners were already revealed from the data, such as working tasks/responsibilities, low rewards, and little prestige. Additionally, social hierarchy within the health care system and social/community perspectives on General Practitioners were common reasons for not choosing General Practitioner as a career pathway.^9^ This study also found that society has high expectations for health workers, not only for the quality of their services but also in socio-cultural aspects, i.e., doctors should have a high socioeconomic level.

Moreover, a piece of supporting evidence that reflected the community voices in Indonesia was also revealed a low level of trust between patients and primary care doctors.^19^ This mistrust will make working as General Practitioner to be seen as not unfavorable, as indicated in this study. As another of the consequences, it will demotivate students to become General Practitioner.

As students begin to work in the real world, there is the possibility that they will choose General Practitioner as their career pathway since their expected future work environment does not yet motivate them. Responses also showed that working status, in-dependency-responsibility, research opportunity, networking, serving community, and career development are not the main factors that will influence them to determine their works’ specialty, as well-described in previous studies.^6^^,^^7^ However, there is still homework to do since the available rewards should be better defined for General Practitioners to upgrade the current image and role of General Practitioner within the present health care system.

Additionally, from an educational point of view, exposure in rural/remote areas tends to have a positive influence on students to choose General Practitioner as their career pathway.^7^ Furthermore, exposure to good-quality General Practitioners could provide the needed role models for students and positively influence them to choose General Practitioner as their career pathway.^20^ Fortunately, most of the students still see General Practitioners as good role models, and only a few made negative comments about General Practitioners. This positive perception should be maintained from the beginning of their education until the completion of their study since it will be externally integrated with the students’ motives for their career choice.^11^^,^^12^

Further research regarding developing a good educational system in nurturing students’ motivation for becoming General Practitioners should be generated through either community-based education or community-oriented education. Furthermore, further study is to respect and listen closely to the voices from the community regarding the primary care system in Indonesia. As consequence, a wider - more comprehensive scope of primary care research is still needed in order to develop a better universal coverage system.

## Conclusions

In a country without a further developed career pathway as a General Practitioner, like in Indonesia, choosing General Practitioner as a firm career is still not preferable for most medical students. Educational strategies could promote General Practitioner as a viable career pathway in order to provide a sufficient number of qualified General Practitioner for Indonesian people by providing good role models of General Practitioners and exposing students to remote/rural areas. Meanwhile, a more positive environment within the health worker community should be established in order to maintain the current system, i.e., helping the General Practitioners maintain the health care system to work effectively as an essential care coordinator within the system and providing proper rewards for General Practitioner for their delivered services.

### Acknowledgments

We are thankful for Prof. Tri Nur Kristina and the UKMPPD team of the national exit exam who gave opportunity for this study to distribute the online questionnaire.

### Conflict of Interest

The authors declare that they have no conflict of interest.
